# Facilitators and barriers to reducing chemotherapy for early-stage breast cancer: a qualitative analysis of interviews with patients and patient advocates

**DOI:** 10.1186/s12885-022-09189-w

**Published:** 2022-02-04

**Authors:** Courtney Andrews, Timothy C. Childers, Kimberly D. Wiseman, Valerie Lawhon, Stacey Ingram, Mary Lou Smith, Antonio C. Wolff, Lynne Wagner, Gabrielle B. Rocque

**Affiliations:** 1 College of Arts and Sciences, University of Alabama, 1720 2nd Avenue South, Birmingham, Alabama 35294 USA; 2 University of Alabama School of Medicine, 1670 University Boulevard, Birmingham, AL 35233 USA; 3 Wake Forrest School of Medicine, Bowman Gray Center for Medical Education, 475 Vine St, Winston-Salem, NC 27101 USA; 4 Research Advocacy Network, Park Blvd, Suite 305, Plano, TX 6505 W75093 USA; 5 The Johns Hopkins University School of Medicine, 733 N Broadway, Baltimore, MD 21205 USA

**Keywords:** Breast cancer, De-implementation, De-escalation, Facilitators, Barriers, Patient perspectives, Covid-19

## Abstract

**Background:**

As the combination of systemic and targeted chemotherapies is associated with severe adverse side effects and long-term health complications, there is interest in reducing treatment intensity for patients with early-stage breast cancer (EBC). Clinical trials are needed to determine the feasibility of reducing treatment intensity while maintaining 3-year recurrence-free survival of greater than 92%. To recruit participants for these trials, it is important to understand patient perspectives on reducing chemotherapy.

**Methods:**

We collected qualitative interview data from twenty-four patients with Stage II-III breast cancer and sixteen patient advocates. Interviews explored potential barriers and facilitators to participation in trials testing reduced amounts of chemotherapy. As the COVID-19 pandemic struck during data collection, seventeen participants were asked about the potential impact of COVID-19 on their interest in these trials. Interviews were audio-recorded and transcribed, and researchers used qualitative content analysis to code for dominant themes.

**Results:**

Seventeen participants (42.5%) expressed interest in participating in a trial of reduced chemotherapy. Barriers to reducing chemotherapy included (1) fear of recurrence and inefficacy, (2) preference for aggressive treatment, (3) disinterest in clinical trials, (4) lack of information about expected outcomes, (5) fear of regret, and (6) having young children. Facilitators included (1) avoiding physical toxicity, (2) understanding the scientific rationale of reducing chemotherapy, (3) confidence in providers, (4) consistent monitoring and the option to increase dosage, (5) fewer financial and logistical challenges, and (6) contributing to scientific knowledge. Of those asked, nearly all participants said they would be more motivated to reduce treatment intensity in the context of COVID-19, primarily to avoid exposure to the virus while receiving treatment.

**Conclusions:**

Among individuals with EBC, there is significant interest in alleviating treatment-related toxicity by reducing chemotherapeutic intensity. Patients will be more apt to participate in trials testing reduced amounts of chemotherapy if these are framed in terms of customizing treatment to the individual patient and added benefit—reduced toxicities, higher quality of life during treatment and lower risk of long-term complications—rather than in terms of taking treatments away or doing less than the standard of care. Doctor-patient rapport and provider support will be crucial in this process.

**Supplementary Information:**

The online version contains supplementary material available at 10.1186/s12885-022-09189-w.

## Background

In the relentless quest to improve survival outcomes, breast cancer treatment has trended toward adding novel targeted therapies to a standard chemotherapy regimen. While the additive benefit of these additional therapies remains unclear, combining multiple systemic and targeted therapies is associated with severe adverse side effects and long-term complications in health [1]. Significant advances in prognostication and treatment provide opportunities to reduce treatment intensity for women with early-stage breast cancer (EBC) in hopes of avoiding treatment-related toxicities without sacrificing efficacy. This is a form of de-implementation, defined as “reducing or stopping the use of a health service or practice provided to patients” [2]. De-implementation of low-value chemotherapy for patients with EBC has become a compelling prospect for oncologists, [3] and clinical trials are underway to test if standard chemotherapy can be de-implemented while maintaining 3-year recurrence-free survival of greater than 92% [4]. However, due to the established medical standard and general expectation of aggressive treatment, it is anticipated that resistance to reducing chemotherapy will be strong at the patient level as well as at the provider, institutional, and societal levels [5]. Effectively facilitating a paradigm shift in cancer treatment from a preference for maximizing treatment intensity to reducing treatment intensity requires understanding and support from stakeholders at all four levels. On the continuum of factors influencing the de-implementation process, Norton et al. (2019) suggest identifying barriers and facilitators to de-implementation prior to developing strategies for clinical practice [2].

Our cross-sectional survey of women with breast cancer found that 58% of respondents would have been interested in participating in a clinical trial testing less than standard chemotherapy, the primary motivating factor being to avoid physical toxicity [6]. Remaining survey respondents expressed unwillingness to participate in such a trial, primarily due to fear of recurrence. In order to explore these findings further and gain insight from patients, we recruited a different sample of EBC patients and patient advocates to participate in a semi-structured interview in which they were asked about their perspectives on de-implementation of chemotherapy. In this paper, we present the results from the interview, focusing on reasons participants identify for being interested or uninterested in participating in a clinical trial testing a less than standard amount of chemotherapy.

The interview was designed to engage EBC patients and patient advocates in a discussion of their willingness to participate in a clinical trial of reduced chemotherapy, what they think will be the most common facilitators and barriers to reducing treatment among newly diagnosed patients, and how they anticipate patients will weigh the costs and benefits of this approach depending on their perception of risk, their relationships with their healthcare providers and their life circumstances. As COVID-19 struck during the middle of data collection, we incorporated a question in the final interviews (*n* = 17) about how the pandemic might alter perceptions and behaviors regarding treatment choice, specifically receiving less chemotherapy.

## Methods

### Participant sample

Qualitative interview data were collected from patients with Stage II-III breast cancer (*N* = 24) receiving treatment at the University of Alabama at Birmingham and patient advocates (*N* = 16). Eligible patients were identified using medical oncology clinic lists, and interviews were conducted from October 2019 to May 2020. The researcher coordinator approached eligible participants during clinic or contacted them by phone. After consenting participants, she collected their demographic information and Shared Decision Making (SDM) preferences. All participants were 18 years or older, able to read and speak English, able to provide informed consent, and deemed appropriate for interview by their medical oncologist. In order to recruit a similar number of Black and White patients, we reviewed patients’ charts to determine racial and ethnic identification. Additionally, a convenience sample of patient advocates was identified through advocacy organizations (Eastern Oncology Cooperative Group, Translational Breast Cancer Research Consortium, Patient Advocate Foundation [PAF], CancerCare, Forge) Advocates were invited by e-mail to participate in the study. While patients are primarily reflecting on their own perspectives and experiences, patient advocates have valuable insight as a result of being part of the decision-making process for many patients in addition to having gone through the process themselves. This study was approved by the University of Alabama at Birmingham (UAB) Institutional Review Board (IRB #170,518,009). Written and electronically signed informed consent was obtained for all participating patients and advocates. Patient participants were incentivized with a $50 gift card at interview completion.

### Data collection

Following the de-implementation framework developed by Norton, Chambers, and Barnett (2019) [7], we define barriers and facilitators as “those elements that may either facilitate or hinder de-implementation efforts.” This analytic framework assesses the extent to which patients would welcome or react adversely to reducing or stopping chemotherapeutic treatment. Our interview was designed to explore in depth what kinds of considerations go into the decision-making process regarding the option to reduce chemotherapy as well as how patients and advocates evaluate these concerns against one another.

The interview questions were developed by the research team specifically for this study (see Supplementary Material). Demographic information collected for each participant included age, racial identity, marital status, zip code Area Deprivation Index (ADI), rural or urban commuting area, treatment duration, participation in a clinical trial, and decision-making preference (collected using a Likert scale ranging from “I prefer to make the final selection about which treatment I will receive” to “I prefer to leave all decisions regarding my treatment to my doctor”). We used an exploratory-descriptive qualitative research design, a methodological framework best suited for the exploration of understudied topics in healthcare research [8], such as de-implementation of low-value chemotherapy. This design uses purposeful sampling and focused interviews to explore how participants are thinking and feeling about a particular subject matter. Interviews were arranged at a time and place convenient to participants, anonymity was ensured, and interviews were recorded for accurate and verbatim transcription. Semi-structured interviews were conducted in person or by phone by a medical oncologist with a background in health services research. Importantly, the interviewer was not involved in treatment decisions for any of the participants interviewed, nor were treating physicians’ names used during the interview. The interview began with an explanation about why the participant had been asked to participate, the purpose of the study, and the objective of the interview – to hear the participant’s ideas and learn about their experiences with chemotherapy. The interviewer then re-confirmed that the participant was comfortable being recorded and asked if they had any questions. The interviewer avoided asking leading questions, instead asking participants for more information with open-ended prompts such as, “tell me more about that.” Finally, the interviewer concluded each interview by asking if the participant had anything else to add on the subject matter, therefore ensuring that each participant was allowed to speak freely and fully about the topic at hand. Interview questions were designed to explore patient interest in de-implementation study participation, barriers and facilitators to participation, preferred verbiage to describe the concept of de-implementation, and recommendations for future patient engagement. As the COVID-19 pandemic hit the United States in March of 2020, interviews conducted after this time included questions about the potential impact of COVID-19 on perspectives regarding de-implementation of chemotherapy. Interviews were audio-recorded using two digital voice recorders. Recordings were transferred to a password protected file on our drive, at which point they were deleted from the recorders. An independent transcription service (Rev) transcribed the recordings. Interviews lasted an average of 25 min and ranged from 15 to 42 min for both patients and patient advocates. All requirements from the Standard of Reporting Qualitative Research [9] checklist were met (see Supplementary Material).

### Analysis

Using qualitative content analysis, five study team members with medical anthropology, public health, and oncological expertise reviewed transcripts and developed a preliminary open coding scheme based on key topics of focus in the interview guide. Two coders subsequently used NVivo software (QRS International) and Atlas.ti 8 (Scientific Software Development GmbH) to conduct “focused coding,” which included a detailed analysis of themes identified during open coding. For example, the initial coding scheme listed facilitators as a theme. During the intensive coding process, all facilitators mentioned by participants were coded separately in order to determine the number of times a specific facilitator was mentioned and by how many participants. Analysts then combined statements with similar meaning (i.e., “less toxicity” and “fewer side effects”). A third study team member was brought in to resolve any discrepancies during this process. Once all coders felt that thematic saturation had been reached, the saliency of each theme was determined based on the number of participants who mentioned that theme. Finally, exemplary quotes characterizing themes were highlighted, and investigator insights relevant to specific facilitators and barriers were noted.

## Results

### Sample characteristics

Forty women participated in qualitative interviews, including 24 women with early-stage breast cancer and 16 patient advocates (see Table 1). The median age of patients was 57 (range 33–79); 13% of patient advocates and 42% of patients were Black or African American. Most participants preferred a shared decision-making approach to treatment decisions. Fifteen patients (62.5%) lived in urban areas, and fourteen patients (58.3%) lived in zip codes classified as middle or high deprivation on the Area Deprivation Index.

**Table 1:** Qualitative interview participant demographics and clinical characteristics (*N* = 40)

	**Patients**	**Advocates**
	*n* = 24	*n* = 16
	n (%)	n (%)
**Age (median, IQR)**		
Median	57	61
Range	33–79	36–75
**Race**		
Black or African American	10 (41.7)	2 (12.5)
White	14 (58.3)	14 (87.5)
**Highest level of education**		
High School	5 (20.8)	0 (0.0)
Some college/Vocational or Technical School	9 (37.5)	2 (12.6)
College graduate (4 year)	7 (29.2)	5 (31.3)
Master’s degree or Professional degree	2 (8.3)	8 (50.0)
Other	1 (4.2)	1 (6.3)
**Rural/Urban Commuting Area (based on zip code)**		
Rural	2 (8.3)	–
Urban	15 (62.5)	–
Unassigned/unanswered	7 (29.2)	16 (100.0)
**Area Deprivation Index (based on zip code)**		
Low	3 (12.5)	–
Mid	7 (29.2)	–
High	7 (29.2)	–
Unassigned/unanswered	7 (29.2)	16 (100.0)
**Marital status**		
Single, Unmarried, living with significant other	5 (20.8)	3 (18.8)
Married	14 (58.3)	13 (81.3)
**Preference for treatment decision-making style (Control Preferences Scale)**		
Patient-driven decision making	1 (4.2)	0 (0.0)
Patient-driven decision making with provider input	5 (20.8)	9 (56.3)
Shared decision-making	14 (58.3)	6 (37.5)
Physician-driven decision-making with patient input	2 (8.3)	1 (6.3)
Physician-driven decision-making	1 (4.2)	0 (0.0)
Missing	1 (4.2)	0 (0.0)
**Breast cancer diagnosis stage**		
II	16 (66.7)	–
III	8 (33.3)	–
**Breast cancer type**		
ER/PR + HER2-	8 (33.3)	–
ER/PR- HER2 +	11 (45.8)	–
ER/PR- HER2-	5 (20.8)	–

*IQR* interquartile range, *ER* estrogen receptor, *PR* progesterone receptor, *HER2* human epidermal growth factor receptor 2

### Interview themes

“I think some patients will be thrilled. I think other patients will be concerned that they are sacrificing efficacy.”

As the above quote indicates, some participants found de-implementation in cancer treatment to be a compelling option, while others had serious concerns about the efficacy of this approach. Beginning with the factors identified as barriers to reducing chemotherapy, we first consider how participants talk about their concerns. These include fear of recurrence or inefficacy of treatment, the preference for more aggressive treatment, concern about participating in a clinical trial, concern about the lack of solid data on outcomes, fear of regret, and having young children. Next, we consider what might assuage these concerns and compel patients to opt for a less aggressive approach. Facilitators included avoiding physical toxicity, understanding the scientific rationale and feeling informed about the anticipated outcomes based on risk level, trust and confidence in providers, consistent monitoring and the option to increase dosage, fewer financial and logistical challenges, and contributing to scientific knowledge. Our aim is to capture the most commonly expressed sentiments for each barrier and facilitator and to let the participants’ insights speak for themselves. Finally, we explore how perceptions around the option of reducing treatment intensity are being re-shaped by concerns related to the COVID-19 pandemic.

### Barriers

#### Fear of recurrence/inefficacy

The primary factor respondents identified as a barrier to interest in reduced chemotherapy was the fear that “it wouldn’t get it all.” Twenty-five participants (62.5%) mentioned the fear of recurrence and inefficacy as a major hurdle in patients’ willingness to opt for a less aggressive chemotherapy regimen. One patient said, “The main thing is you want to be sure that it’s gone and that it doesn’t come back.” One advocate described the compulsion to do “whatever it takes at that time to make sure that you've covered all your bases and you've got the right treatment.” Fear of recurrence is particularly scary for patients due to the general understanding that any recurrence does not bode well for survival chances. To this end, one advocate said that the first time around, “we need to do everything we can so it doesn't come back, because we know if it comes back then it's treatable but not curable. This is the time of cure.”

#### Preference for more aggressive treatment

Sixteen participants (40%) mentioned the preference for more aggressive treatment as a barrier to participation in a trial of reduced chemotherapy. Respondents that perceived themselves as higher risk were particularly apt to worry about whether or not the reduced approach would be as effective as the standard treatment, and with little evidence to offer clarity about this, patients are more likely to deal with this uncertainty by erring on the side of more rather than less chemotherapy. One patient said her preference to do more was based on the fact that she was diagnosed as stage three, explaining, “I was bad enough where I felt like I had to.” However, she said that had she been diagnosed at an earlier stage, she would have considered doing less had that been consistent with her doctors’ recommendation. Another patient said what drove her to do more was her sense of how aggressive the tumor was. Speculating that “if the tumor wasn’t as big, if it hadn’t been in any of the lymph nodes” she may have done a reduced approach, but considering her situation, she said, “I guess it’s better to be safe than sorry.” Another said her decision to do more was based on her “strong family history of breast cancer.” Two African-American respondents mentioned that their preference for more aggressive treatment was primarily driven by their perception of Black women being at higher risk for recurrence, with one suggesting that “because I have a higher death rate from metastatic breast cancer in the African American community, maybe I need more.” One patient said that when she was initially making decisions about her treatment, she was shown research suggesting that less chemotherapy did not lower the chances of a recurrence; however, she said she went with the more aggressive approach as a preventative measure, noting, “It was probably psychological.” Finally, one advocate suggested that doing more was necessary to quell her anxiety, saying, “Well, why wouldn't you do every single thing you could possibly do that was even remotely in your control? From changing your diet, to exercising more, to doing all those things that we feel like give us a little bit of control in a situation where we feel like we have none, why wouldn't I do that?”.

#### Reluctance to participate in clinical trials

Fourteen participants (35%) expressed a general lack of interest in clinical trials of any kind. This sentiment was primarily based around a reluctance to deviate from the standard of care and concern over being what respondents referred to as a “guinea pig” or “lab rat.” One patient said she did not like the feeling of being experimented on, though she qualified this by acknowledging that trials are the only way to learn “what works and what doesn’t.” One participant said that, in general, patients “don't like new, they want the old trusty, what works good and that kind of thing.” One respondent mentioned the logistical burden of participating in a clinical trial, suggesting that treatment will take more time and require patients to come in more often. External pressure from family members was also mentioned as a barrier to participation in trials. One advocate mentioned the stigma related to trial participation, a general misconception of what a trial is, and what the benefits of participating in a trial are. She thought that recruitment would be easier if the general population had more information about clinical trials.

When asked why she would be hesitant to participate in a clinical trial, one African-American patient simply said, “the Tuskegee test.” In speaking about Black women in a general sense, an African-American advocate said preference for a more aggressive approach centers around “a lack of understanding as to what more is. And a lack of participation and research as a process that we're involved in.” She followed up by saying, “and so I come into that decision already not trusting the process. And if I already don't trust the process, then the [de-]escalation sounds suspicious to me.”

#### Lack of information about the expected outcomes of reduced chemotherapy

Thirteen participants (32.5%) mentioned a general sense of uncertainty around treatment that has not been sufficiently tested. One advocate said that when people hear about a study, they think “Ooh, ‘study,’ that means they don't know the answer, yet.” Several respondents agreed that without the data to prove efficacy of reduced treatment, it may be difficult to get patients to participate. When asked about the biggest barrier to generating participation in trials of less chemotherapy, one advocate said, “I think it's the fear of the unknown, which you're facing anyway when you're looking at starting chemotherapy, you don't know exactly what's going to happen.” This fear centers around not having enough data on survival rates with an untested approach. Of the patients who mentioned data on safety/recurrence, eleven said they thought patients would be more willing to do less chemotherapy if there were more data that showed good outcomes for patients with similar types and stages of cancer. “Numbers have great power,” one advocate said. Of the respondents asked specifically about these statistics, all said survival rates in the 90th percentile would give them comfort.

#### Fear of regret

Nine participants (22.5%) said they feared regretting the decision to choose a treatment of less intensity. One advocate said, “I think people are afraid that they will regret their decision, right? You don't really know if it's right or wrong.” Another said, “Even though the chemo almost killed me because of an infection that we didn't know about, it still put me at ease having had the chemo.” Another said, “Well, I think if it came back…you would think ‘Why didn’t I do that?’” Another said, “I know that that's a fine line because chemotherapy has its own issues and stuff, but I'm more of a person where I would just want to go ahead and get as much treatment as I possibly can that is recommended because I don't want to look back and say I should have had more.” She added, “If it came back during a graduation or a wedding or some major life milestone, I'd be kicking myself. I'd be like, ‘I should have taken care of this years ago.’”.

#### Having young children

Eight participants (20%) mentioned preferring more aggressive treatment based on the ages of their children and the desire to see their children grow up. In explaining her own decision-making process, one advocate said, “I mean there was a trial, but in my mind I had to be able to look my kids in the eyes and know I did everything in my power. So if there wasn't research to support the lower dose at that stage—I wouldn’t have done it. I still probably wouldn't right now because my kids are still growing, but if I were in my mid to late 50's and my kids are out of college, yeah that would be something that I would take that small risk.” One advocate suggested that patients need to know that “they left no stone unturned.” One patient talked about her own reluctance in terms of a desperation to live, to survive long enough to see her daughter graduate college and get married. One older patient chose to do a less aggressive approach, which she felt was right for her, but she speculated that a younger woman with small children (she was thinking specifically of her daughter-in-law) might choose to do “anything under God’s green heaven” to stay alive.

### Facilitators

#### Avoiding physical toxicity

When asked what might compel patients to participate in a clinical trial of less chemotherapy, respondents overwhelmingly (*n* = 32, 80%) mentioned avoiding physical toxicities as the primary motivating factor. This included everything from hair loss to fear of death. For example, one patient said, “Chemo is the worst thing that you can do to a human being. And when women, when anyone, hears the word chemotherapy, they're scared out of their life. When I told certain people that I had cancer, they said, ‘Oh, I'm so sorry. Oh, I'm so sorry. Oh, you have to go through chemo.” Another patient said, “Chemotherapy is like the worst experience of my life in any type of treatment. And almost dying in the process of having something that's supposed to be saving me." One patient said, “It feels like the drug is going to kill you before the cancer would.”

In speaking of the rationale behind doing less chemotherapy, one patient said, “Just the toxins you’re putting in your body.” The appeal of reduced toxicity centers around the hope of less down time and a higher quality of life while undergoing treatment and in the long term. This included the ability to continue normal, everyday activities, especially those related to family life and work. As one advocate explained, having a higher “overall quality of life [and the] ability to maintain as much normalcy as you can” would be a motivating factor in opting for the less aggressive route. In thinking about her family situation at the time of treatment, one patient recalled, “My daughter had just turned three, and I would have wanted to have more of a quality with her than be scared I was going to be throwing up and sick all the time.” One advocate told a story about a friend of hers who had chemotherapy but ended up having a transplant. Now, stricken with neuropathy, she feels that “had he gone right to transplant, he would be much better off.” Another advocate said, “I now know that the treatment I had was unnecessary. I could've completely not had chemo. Chemo wasn't fun. It made me sick.”

#### Understanding the scientific rationale/feeling informed about the anticipated outcomes based on risk level

As chemotherapy is the most commonly expected treatment for cancer, understanding the scientific rationale behind offering a less intense approach to chemotherapy is important for patients in making decisions about their treatment. Sixteen participants (40%) said that providers should take great care in explaining this rationale to patients so that they understand the anticipated benefits and outcomes of this seemingly paradoxical approach. For example, one patient said, “I'm the type that it helps me understand why we're doing what and what this is for and what that is for…And then also, when all your friends and family ask what's going on, you can explain it better.” This rationale was most often framed in terms of the short- and long-term side effects of chemotherapy and the hope that reducing chemotherapy will alleviate some of these toxicities but be just as effective as the current standard of care in curing the cancer. For example, one advocate said, “I think a conversation [is needed] about why we are really talking about de-escalation and why it's important. Not just today, tomorrow, but 10 years from now when you can really talk about those long-term side effects, the collateral damage to the patient, on the front end.” Another advocate said, “I think it will be important for you to identify which chemotherapies they'll be getting and which they won't, and what the data says about the eliminated chemotherapies and what they add to the regimen both in terms of benefits and toxicities.”

To this end, fourteen participants (35%) mentioned that feeling informed about all of the options available to them, why the option of less chemotherapy was being presented, and what the expected outcomes were regarding each option would increase their likelihood of agreeing to participate in clinical trials of less chemotherapy. Patient advocates in particular feel very strongly about making sure patients understand their risk level and why doing less might be beneficial for them. One advocate said, “I think as a patient I would rather have you have more information about my cancer than less. More information you can collect about my cancer and how I'm responding, more comfortable I would feel deviating from standard of care.”

Patients appreciate as much information as possible in making choices about their treatment, and they are not always initially aware of the adverse side effects of chemotherapy. One patient commented, “Understanding why I was taking the chemo and what it was going to do and what the reactions that it would do to my body and how I would feel. Things like that really helped.” These explanations may come from providers, advocates, or from members of the community whose treatment is underway and can personally attest to the lived experience of treatment. As one advocate said, “I think as long as the patients truly understand what all of it really means, and if it can be thoroughly explained to them in their terms that they would understand, I truly think they, long-term wise, I think they would be willing to participate.”

#### Trust and confidence in providers

Fifteen participants (37.5%) mentioned putting a high premium on their doctors’ recommendation when it came to making decisions about treatment. Some patients said they were inclined to go with whatever their doctor recommended, while others said they would be willing to consider the doctor’s opinion in the process of making the final decision for themselves. For example, one patient said the doctor’s recommendation “weighs heavy, because they're the expert in the field. I'd like to know what they would do if it was themselves or if it was their wife or their mom. I like to know their personal opinion, as honest as possible. And then I also like to know their scientific opinion and what they see, because they're the experts.”

When asked if patients would worry about getting less chemotherapy, one respondent said, “Not if they trust their doctors.” One respondent said if her doctors had suggested less chemotherapy than the standard of care, she would have wondered, “Are we doing enough? Are we in this fight to win?” However, this woman also said that she would have been willing to try it if her doctors recommended it. One advocate described her extreme apprehension at her doctor’s recommendation to not do chemotherapy but said that she ultimately came around to that decision. One advocate expressed that it is not just about blindly going along with the physician’s recommendation, but that trust and confidence comes from having a relationship and a rapport with the physician and the confidence that the “physician is not just looking at data,” but looking “at the person and [caring] about the person.” One advocate said, “I would think that your research with the physicians buying into it and saying, ‘This is what, with current medical information available to us, we feel this is a viable option for your treatment and you'll have potential to have good results.’” The doctor’s confidence in this approach—even without the hard data—is very important to patients.

#### Consistent monitoring/option to increase dosage

Ten participants (25%) cited the assurance of being monitored regularly and the option to increase the dosage if necessary as an incentive to participate in a trial of less chemotherapy. One advocate said that she thinks patients would be less fearful as long as “they're made to feel safe and secure and monitored.” Another noted that patients may not readily assume this option is available to them but that it can be a compelling factor if it is presented early and explained clearly. Again, it is important for patients to know their options and to feel cared for at each stage of the treatment process.

#### Fewer financial and logistical challenges

Nineteen participants (47.5%) mentioned that financial concerns would factor into their decisions about reducing treatment. Reducing the amount or duration of chemotherapy is associated with fewer co-pays, the ability to keep working, lower travel costs, and fewer out of pocket expenses related to treatment. One patient said, “If it puts you in a physical bind where you can't afford the treatment, or if the treatment that you are taking just makes you so sick and feel horrible, then you do think; well, is this the right route for me to take, or should I be less aggressive?” One patient mentioned that with regard to her own treatment, finances were “not taken into consideration because it was my life.” However, she did acknowledge that living paycheck to paycheck while undergoing chemotherapy is quite burdensome. Another said finances were not a big concern for her, but acknowledged that “if people have limited income, it probably would if they don't have a good cancer policy.” These concerns largely depend on patients’ financial situation at the time of diagnosis. As one advocate said, “that's such a personal thing, because that three months [of treatment] could be a very different scenario for someone who has to be at work, and who can't pay their mortgage if they're out of work for three more months getting chemo and all of those things.”

Logistical concerns also factored into patients’ considerations and would incline them toward a less aggressive approach. One advocate mentioned the logistics of “coordinating with family and caregivers around their daily treatment schedule.” In this scenario, less treatment time means less strain on the patient and their families. Another said, “If it's less frequent than most of the regimens or a different schedule that allows them to spend less time or have less side effects that are impacting their daily life.”

#### Contributing to scientific knowledge

Four respondents (10%) mentioned the importance of trials in the research process as a facilitator to doing a trial of less chemotherapy. One patient said, “I would be more than happy to try because that's the only way to know if a treatment plan is going to work—through trials, I mean somebody has to do it to know that it's going to work.” Another said, “I had a lot of family and friends that were worried that I was going to be a ‘guinea pig,’ but I was kind of excited to be a part of a clinical trial, especially finding out that not many people get that opportunity and that we don't have that many cancer centers, even in the nation at all. So I felt privileged to be a part of it.” One advocate mentioned participation in a clinical trial of less chemotherapy as an opportunity to help others in the future. She said, “The only way we now know that we can do less with the same results is through these trials. That's what I think we would need to tell patients because the thing that I kept telling myself when I was able to be on this study was, ‘You may not be cured, but you will help someone else. You're doing something to help someone else.’ That appealed to me at the time. It gave me some hope beyond myself.”

### Impact of COVID-19 on interest in reducing chemotherapy

“[Patients are] anxious because they're in the hospital room…even though they're getting the treatment that they need, there's an anxiety, so they're trying to play off this risk of infection and risk of cancer coming back in their minds and it's very complex.”

When the COVID-19 pandemic hit the United States in March of 2020, we decided to add a question about how participants think the pandemic will impact patients’ decisions regarding cancer treatment, specifically, if they think patients may be more inclined to do less chemotherapy considering the current situation. Of the seventeen participants who were asked, seven (41.2%) stated that when they were making decisions about their treatment originally, they would not have been interested in a less aggressive approach, and one said she was unsure. Of these, only one said she would stick with more aggressive chemotherapy under the current circumstances. She clarified, however, that she would take extra precautions in her life to avoid contracting the virus while undergoing chemotherapy.

The primary reason cited for this change was that chemotherapy compromises the immune system. One patient said, “Thank goodness I am not having to go through that right now because that would have really caused me to have a lot of anxiety to know that my immune system was compromised because of the chemo and then facing all the concerns with the corona.’ So I would think that people would be a little less is more with that because you're opening yourself up to being susceptible to the corona.” Another said, “I would be worried about the treatment because…this virus…attacks you quicker when your immune system is low, and so that would be a concern of mine.”

Several patients also cited the benefit of reduced exposure due to less time spent at the clinic. In speaking about going to appointments, one patient said, “I wouldn't worry about getting the chemo as much as I would worry about getting the virus.” Another said, “Just that fear of if you did need to be receiving treatments, could I get in and out of the clinic safely? Not necessarily would I be safe during the treatment, but you've got to walk through those halls to get there.”

## Discussion

De-implementation of low-value chemotherapy in cancer care requires “buy in” from patients and providers and good communication between the two. Our aim has been to elicit and explore the perspectives of patients and advocates regarding the facilitators and barriers to reducing chemotherapy for EBC patients. This is important for two reasons. First, in order to generate enough data to determine whether or not standard treatment regimens that combine multiple chemotherapies are, in fact, unnecessary in terms of added value, clinical trials must include a wide sampling of the general population. When the long-standing expectation in cancer treatment is to do more, not less, recruiting participants to these trials may be difficult. Our study only scratches the surface of how perspectives on barriers and facilitators to reducing chemotherapy differ by race, education, socio-economic status and life circumstance. Understanding how patients of different backgrounds and situations assess and weigh the concerns and incentives of such a trial are necessary in developing patient-centered language to mitigate the barriers in the pursuit of less invasive and yet highly efficacious treatment regimens. This is particularly pertinent in the context of COVID-19, as patients may be more inclined to do less in order to reduce their risk of exposure to the virus and to avoid further compromising the immune system.

Second, this knowledge will help incorporate new forms of treatment into cancer care more widely. Paradigm shifts in cancer treatment (and healthcare generally) can be difficult to implement even when there is sufficient evidence to suggest their superiority [10]. As one of our participants stated, there is a psychological compulsion to do more even when chances of survival are the same with a less aggressive approach. Studies confirm that low-value practices continue to be utilized in care settings even though they are associated with high costs and little to no improvement in patient outcomes [11]. For example, mastectomy is still commonly practiced even though there is no difference in recurrence rates compared to lumpectomy plus radiation treatment [12]. To this end, one study found that “extra-medical influences” played a more compelling role than evidence in patient choice to undergo radical mastectomy [13]. While data-driven research on patient outcomes is important, de-implementation efforts must look beyond—and *before—*outcomes data to understand what is driving the continued utilization of low-value, high-cost services.

Our results demonstrate that patients weigh a complicated set of considerations in making decisions about their cancer treatment. On one hand, fear of recurrence is the primary rationale for choosing a more intense chemotherapy regimen that includes additional targeted therapies. This may be especially true for patients that have little familiarity with the adverse side effects of chemotherapy. This is consistent with other research on cancer treatment that shows fear of recurrence and perceived survival benefit are the primary motivators in wanting to treat as aggressively as possible [14]. However, this fear may be somewhat attenuated by the prospect of fewer toxicities and long-term health complications associated with less aggressive forms of treatment. Since efficacy data for de-implementation is not yet available, it is important for patients to understand the rationale of reducing chemotherapy and to feel confident in their providers’ support of this approach. Patients put a high premium on feeling like their treatment course is tailored to their risk level, their unique bodies and tolerances, and their life circumstances. The comfort of being monitored closely throughout treatment and able to switch course if necessary is a compelling factor for many patients to take a less aggressive approach initially.

### Strengths and limitations

This study has several limitations. First, only female patients with breast cancer were interviewed, which may not reflect the views of males or patients with other cancers. Second, the interview only asked about the de-implementation of chemotherapy, not other forms of treatment, such as radiation or surgery. Third, the geographic setting of the interviews was one cancer center in Alabama, which may limit the generalizability of the themes mentioned by participants. However, themes were consistent with those from the initial survey, which surveyed a nationally representative sample. Fourth, the person who conducted the interviews is a medical oncologist, which may have created somewhat of an unbalanced dynamic with participants, particularly patients. Finally, while we interviewed nearly an equal number of Black and White patients, we were less successful in recruiting Black patient advocates, whose opinions and perspectives on the decision-making process for Black women may be particularly pertinent because they are likely to work with a larger number of Black patients.

Regarding the strengths of the study, we are pleased to contribute to the emerging body of work exploring facilitators and barriers to de-implementation efforts in cancer treatment. Little research has called for an in-depth exploration of patient perspectives and preferences as they weigh decisions about chemotherapy. Our participants provided us with critical insight into why reducing chemotherapy is a compelling option for some people and something others will not even consider. Understanding the experience and anticipating the needs of patients during initial diagnosis, the treatment decision-making process, treatment, and post-treatment care are important elements of clinical trial design. Understanding nuances and discrepancies in facilitators, barriers and preferred language among patients and being able to respond to those will assist researchers and providers in providing the best care to patients.

## Conclusions

Based on our findings, in order to get as much patient “buy in” as possible, we recommend that de-implementation strategies and recruitment be framed in terms of customizing treatment to the individual patient and added benefit—reduced toxicities, higher quality of life during treatment and lower risk of long-term complications—rather than in terms of taking treatments away or doing less than the standard of care. Doctor-patient rapport and physician recommendation will be a crucial part of this equation, so future research should focus on physician perspectives on reducing chemotherapy. Because the Covid-19 pandemic has created a different set of concerns and shifted the conversation in cancer care, the current context might provide a ripe opportunity for fostering support for de-implementation efforts in cancer treatment.

## Supplementary Information

**Additional file 1. **Patient and Patient Advocate Interview Guide Phase III:

**Additional file 2.** Standards for Reporting Qualitative Research (SRQR)*.

## Data Availability

The raw data collected for this study is available from the corresponding author upon reasonable request. We do not wish to make this publicly available at this point because we are still conducting analysis of the data and writing up the results.

## Abbreviation

EBC: Early-stage breast cancer
