# Supplementary material for: Facilitators and barriers to reducing chemotherapy for early-stage breast cancer: a qualitative analysis of interviews with patients and patient advocates
Source: BMC Cancer. 2022 Feb 4;22:141. doi: 10.1186/s12885-022-09189-w (PMC8815019; doi:10.1186/s12885-022-09189-w)
Supplement: Supplementary file 1 — Additional file 1. Patient and Patient Advocate Interview Guide Phase III: [file 12885_2022_9189_MOESM1_ESM.docx]

**Patient and Patient Advocate Interview Guide Phase III:**

Good [morning/afternoon/evening.]. My name is [TBN] and I will be conducting today’s interview. You have been invited to participate in this interview because you have a history of breast cancer.

We are following currently planning a study called COMPASS, which test the ability to decrease the amount of chemotherapy given to patients with Her2-positive breast cancer. This may result in fewer short-term or permanent side effects from chemotherapy. The focus of this study is for patients who have no cancer remaining at surgery (either in the breast or underarm lymph nodes) after treatment with 12 weeks of a single chemotherapy and two HER-targeted therapies. We will evaluate if these patients have recurrence rates that are similar to rates reported in prior studies with multiple chemotherapy drugs plus two HER2 targeted therapies, which is the usual approach for patients not in a study.

For today’s discussion, we are interested in hearing from you about what your opinions decreasing the amount of chemotherapy for patients with breast cancer and how to talk to patients about participating in a trial of less chemotherapy that is usually given. Please know that there are no right or wrong answers here today. We want to understand different experiences and perspectives on this treatment approach. Please be open and candid with us. Please attempt to avoid using your name or your doctors’ names during the interview.

Before we start, I want to tell you that everything you say during your conversation with me today will remain confidential. Your responses will not be shared with your doctors or anyone outside the research team. We may use data, without your name or other information by could identify you, in quality improvement reports and publications. Would it be OK if I record our conversation so I can best capture your responses to these questions?

Do you have any questions before we begin?

1. Please tell me how you think you would have felt about participating in a study where you received less than the usual amount of chemotherapy? How would you feel if you received less than the usual amount as a new standard of care?

(open ended, allow them to tell their story if desired)

1. What do you think would be potential barriers to participating in a trial like this one?

Probes:

1. Fear of recurrence
2. Worry about regret
3. Lack of interest in getting something not “standard-of-care”
4. Focus on doing less instead of more
5. What would have made you more comfortable participating in a trial like this? Or receiving less chemotherapy as standard of care?
   1. Being told you have lower risk because of your response to treatment
   2. Avoidance of physical toxicity
   3. Logistical benefits (ability to work)
   4. Financial concerns
6. How would you feel during or when you finished treatment if you heard your friends with similar cancer received more treatment than you?
7. We are interested in what words we can use to describe this approach. What words would you use to describe getting less chemotherapy?
   1. De-escalation
   2. De-intensification
   3. Optimization
8. How would you recommend talking to patients about participating in a trial of less chemotherapy?
   1. How can we ensure patients understand the treatment approach (transparency)?
   2. How can we avoid causing fear?
   3. When should we talk to patients about this approach of reducing treatment? baseline, during treatment, at surgery
   4. How did you thinking about recurrence change during treatment?

Thank you very much for taking the time to participate in this study.
